# Evidence of Destabilization of the Human Thymidylate Synthase (hTS) Dimeric Structure Induced by the Interface Mutation Q62R

**DOI:** 10.3390/biom9040134

**Published:** 2019-04-03

**Authors:** Cecilia Pozzi, Ludovica Lopresti, Matteo Santucci, Maria Paola Costi, Stefano Mangani

**Affiliations:** 1 Department of Biotechnology, Chemistry and Pharmacy—Department of Excellence 2018-2020, University of Siena, 53100 Siena, Italy; lopresti4@student.unisi.it; 2 Department of Life Sciences, University of Modena and Reggio Emilia, 41125 Modena, Italy; matteo.santucci86@gmail.com (M.S.); mariapaola.costi@unimore.it (M.P.C.)

**Keywords:** human thymidylate synthase, interface variant, dimer destabilization, circular dichroism, thermal stability, X-ray crystallography, site-directed mutagenesis

## Abstract

In human cells, thymidylate synthase (TS) provides the only source of 2′-deoxythymidyne-5′-monophosphate (dTMP), which is required for DNA biosynthesis. Because of its pivotal role, human TS (hTS) represents a validated target for anticancer chemotherapy. Nonetheless, the efficacy of drugs blocking the hTS active site has limitations due to the onset of resistance in cancer cells, requiring the identification of new strategies to effectively inhibit this enzyme. Human TS works as an obligate homodimer, making the inter-subunit interface an attractive targetable area. Here, we report the design and investigation of a new hTS variant, in which Gln62, located at the dimer interface, has been replaced by arginine in order to destabilize the enzyme quaternary assembly. The hTS Q62R variant has been characterized though kinetic assay, thermal denaturation analysis and X-ray crystallography. Our results provide evidence that hTS Q62R has a reduced melting temperature. The effective destabilization of the TS quaternary structure is also confirmed by structural analysis, showing that the introduced mutation induces a slight aperture of the hTS dimer. The generation of hTS variants having a more accessible interface area can facilitate the screening of interface-targeting molecules, providing key information for the rational design of innovative hTS interface inhibitors.

## 1. Introduction

Thymidylate synthase (TS, EC 2.1.1.45) plays a pivotal role in human cells, since it catalyzes the reductive methylation of 2′-deoxyuridine-5′-monophosphate (dUMP) to 2′-deoxythymidyne-5′-monophosphate (dTMP), using N^5^,N^10^-methylenetetrahydrofolate (mTHF) as cofactor. According to the reaction mechanism, the thiol of the catalytic Cys195 attacks the carbon atom in position 6 (C6) on the dUMP pyrimidine, forming a covalent adduct (Figure S1) [1]. The dUMP uracil carbon in position 5 (C5) is thus activated to accept the methyl moiety (C11) and the hydride donated by mTHF. In human cells, TS provides the only synthetic source of dTMP necessary for DNA biosynthesis, indeed its inhibition halts the replication processes and induces apoptosis in rapidly dividing cells, an effect known as “thymineless death” [2]. This classifies human TS (hTS) as an important target in anticancer chemotherapy. As a matter of fact, various TS inhibitors targeting the enzyme active site such as FdUMP (the active metabolite of 5-fluorouracil) and raltitrexed, are currently in use as anticancer agents [3,4]. Nonetheless, the use of these classical hTS inhibitors has restrictions due to the onset of resistance induced by TS overexpression [3,4,5]. Thus, new inhibition strategies have to be explored to effectively inhibit hTS without causing resistance in cancer cells.

**Figure 1:**
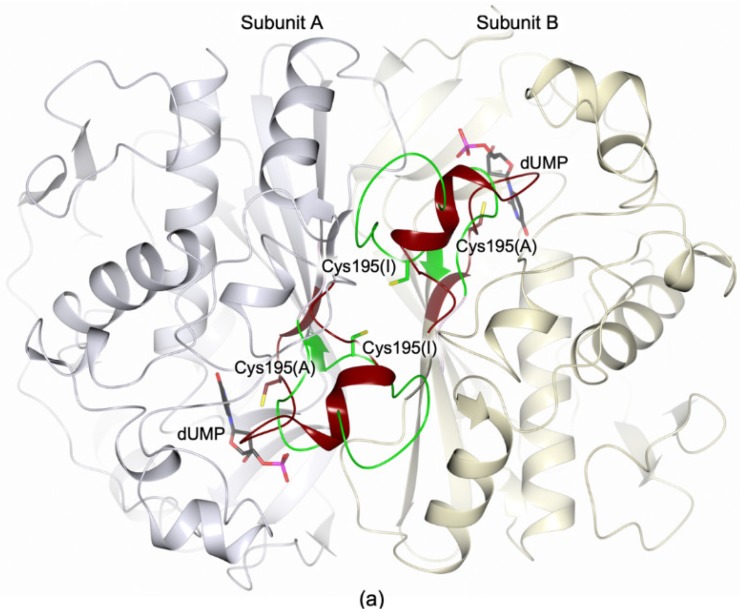

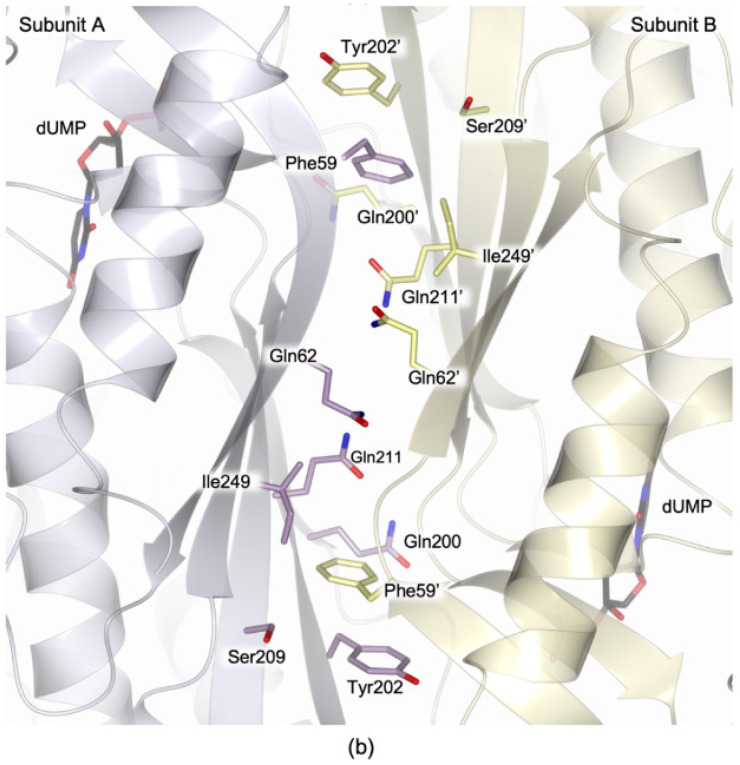
(**a**) Cartoon representation of the superimposition between the human thymidylate synthase (hTS) homodimer (subunits A and B are colored light lilac and yellow, respectively) in the active (Protein Data Bank, PDB, id 5X5D [13]) and inactive (PDB id 3N5G [14]) conformations. The two orientations of the catalytic loop (residues 181–197), defining the active (brown trace; PDB id 5X5D [13]) and inactive (green trace; PDB id 3N5G [14]) conformations, are displayed. The catalytic cysteine is shown in sticks in the active (A) and inactive (I) conformations (brown and green carbons, respectively). The position of the catalytic cavity is indicated by the presence of the substrate 2′-deoxyuridine 5′-monophosphate (dUMP, in sticks, black carbons; PDB id 5X5D [13]). (**b**) Interface view of the two Phe59 pockets, proved to be important for enzyme dimerization [12]. The position of the nearby Gln62, facing Gln62′ on the cognate subunit, is shown. Residues are displayed in sticks (carbon atoms are color-coded according to the parent subunits). In all figures, nitrogen atoms are colored blue, oxygen red, sulfur yellow, and phosphorous magenta.

Former structural studies have revealed that the hTS homodimers are able to switch between two alternate conformations, named active and inactive, primarily by changing the orientation of the loop including the catalytic Cys195 (catalytic loop, residues 181–197; Figure 1a) [6,7]. In the active conformation, Cys195 is exposed inside the catalytic cavity, whereas it is moved at the dimer interface upon the switch to the inactive form. The transition to the active conformation is fundamental to create the functional active site in which dUMP is accommodated, preceding the binding of cofactor (Figure S2) [7]. Inside cells, hTS acts also as a regulatory protein by binding RNAs, including its own mRNA (TSmRNA) [5,8]. It has been proposed that active site-targeting inhibitors of hTS stabilize the active conformation of the enzyme that reduces the affinity of TS for the TSmRNA [5]. This removes the translational arrest and triggers TS overexpression, leading to drug resistance [5]. The TSmRNA binding site on hTS is yet uncharacterized. Even so, there is evidence that the hTS dimer interface plays an important role in hTS-mRNA recognition, perhaps by controlling conformational transitions that alternatively expose and hide the TSmRNA recognition site [9,10,11]. The hTS dimer interface represents an attractive targetable area for the development of new inhibitors that could avoid the onset of drug resistance. Nonetheless, the generation of interface-targeting molecules is tricky and the identification of targetable spots on the interface area is a fundamental requisite. For this purpose, we formerly probed the hTS dimer interface through the generation of a set of alanine variants [12]. Among them, the mutant F59A showed a meaningful dimer destabilization effect, resulting in a K_d_ of 10 (±2) × 10^−5^ M, more than three orders of magnitude higher than that measured for the wild-type hTS, K_d_ of 5 (±1) × 10^−8^ M (both determined through fluorescence resonance energy transfer, FRET, based assays) [12]. This significant gain in the dissociation constant was interpreted as a shift of the monomer-dimer equilibrium towards the monomeric form [12]. This evidence further suggests that the area surrounding Phe59 (Figure 1b) is important to stabilize the enzyme quaternary structure, classifying it as a potentially druggable spot for the development of interface-targeting inhibitors.

On the basis of these results, we have introduced a bulky, charged residue at the dimer interface close to Phe59, in order to generate destabilized homodimers that can facilitate the access to this area, simplifying the identification of interface-binding molecules. The hTS variant Q62R, having the interface residue Gln62 replaced by arginine, was generated and characterized through kinetic assay, circular dichroism (CD) thermal denaturation analysis and X-ray crystallography. Residue 62 is proximal to Phe59 and it faces itself on the cognate subunit enhancing the electrostatic repulsion effects induced by the introduction of a charged residue in this position (Figure 1b).

The CD thermal denaturation studies, performed on the wild-type enzyme and the Q62R variant, showed meaningful variations in the melting temperature of the mutant, evidencing a destabilization effect induced by the introduction of Arg62 at the dimer interface. The structure of hTS Q62R was determined and compared to that of the wild-type enzyme, highlighting a slight aperture of the mutated enzyme homodimer. Furthermore, the structural analysis revealed that the variant adopts the active conformation, entrapping a cofactor analogue molecule within the catalytic cavity. The kinetic characterization of hTS Q62R is consistent with the crystallographic evidence. Our results show that the mutation Q62R effectively destabilizes the hTS homodimer, supporting the importance of this area for the enzyme quaternary assembly.

## 2. Materials and Methods

### 2.1. Cloning and Site-Directed Mutagenesis

The hTS variant Q62R was generated by site-directed mutagenesis using partially overlapping primers (forward primer: GCATGCGGGCGAGATATTCATTAC; reverse primer: CTCGCCCGCATGCCGAAGACGC; purchased from Sigma-Aldrich, Milan, Italy) and the pQE80L-hTS plasmid (including the gene coding sequence for hTS cloned within the BamHI–HindIII restriction sites) as template. The 50 µL PCR reaction mixture included 50–100 ng template DNA, 1 µM primer pair, 200 µM dNTPs and Expand High Fidelity (Roche Biochemicals, Basel, Switzerland). The reaction was performed through an initial denaturation step (94 °C, 5 min), followed by 25 cycles of denaturation (94 °C, 1 min), annealing (52 °C, 1 min), and expansion (72 °C, 10 min), and by a final extension step (72 °C, 20 min). Afterwards, an aliquot (10 µL) of the reaction mixture was incubated overnight at 37 °C with 1 µL of DpnI (NEB, Ipswich, MA, USA). The resulting sample was heat-shock transformed in the *E. coli* TOP10 strain and positive transformants were selected on LB-Agar plates supplemented with 100 mg L^−1^ ampicillin. A single colony was cultured in LB medium added by 100 mg L^−1^ ampicillin (14 h, 37 °C, 220 rpm) and used for plasmid extraction (EZNA DNA extraction kit I, Omega Bioteck, Norcross, GA, USA). Site-directed mutagenesis was confirmed by sequencing the entire TS gene (the absence of unwanted mutations was also verified; sequencing service performed by Eurofins, Ebersberg, Germany).

### 2.2. Protein Expression and Purification

The wild-type hTS was expressed as His^6^-tag protein (HT-hTS, the non-cleavable N-terminal His^6^-tag was encoded by the pQE80L expression plasmid) in the *E. coli* strain BL21(DE3) as previously described [12], with minor modifications. Briefly, bacteria were cultured at 30 °C in the auto-induction medium ZYP-5052 [15] for 30 h. Cells, harvested by centrifugation (3000 g, 15 min, 8 °C), were resuspended in buffer A (50 mM HEPES, pH 7.5 and 30 mM NaCl), added by 20 mM imidazole, 0.2 mM phenylmethylsulfonyl fluoride (PMSF) and 0.5 mg mL^−1^ lysozyme, and then disrupted by sonication after 60 min incubation on ice. The cell-free extract, obtained by centrifugation (12,000 g, 60 min, 8 °C), was applied to a HisTrap HP 5 mL column (GE Healthcare, Milan, Italy) and eluted using a step-gradient protocol, by applying 250–500 mM imidazole concentration in the same buffer. Fractions containing the target protein were pooled and dialyzed in buffer A. The resulting sample was concentrated (Vivaspin 20 molecular weight cut-off 10 kDa, Sartorius, Göttingen, Germany) and further purified by size exclusion chromatography on a HiLoad 16/600 Superdex 75pg column (GE Healthcare, Milan, Italy). The elution profile was consistent with the enzyme dimer assembly (not shown). The high purity (>98%) of the resulting protein sample was confirmed by SDS-PAGE analysis (NuPAGE 4-12% Bis-Tris protein gels; Thermo Fisher Scientific, Waltham, MA, USA) and MALDI-TOF mass spectrometry (Toscana Life Science, Siena, Italy).

Attempting to produce the HT-hTS Q62R variant under the condition optimized for the wild-type enzyme resulted in an almost complete localization of the target protein in inclusion bodies. Therefore, a wide set of expression conditions was screened by testing different culturing media (Luria Broth, Super Broth, ZYP-5052), incubation temperatures (20 and 30 °C), inductor concentrations (isopropyl β-D-thiogalactopyranoside, IPTG, 0.1 and 0.5 mM), and incubation times (20 and 48 h). Our best condition turned out to be by culturing bacteria (plasmid transformants of *E. coli* BL21(DE3)) in the auto-induction medium ZYP-5052 [15] at 20 °C for 48 h. The HT-hTS Q62R variant was purified by nickel-affinity and size exclusion chromatography following the same procedure described for the wild-type enzyme.

### 2.3. Enzymatic Activity Assays

Enzyme activity assays were performed spectrophotometrically, according to a reported protocol [12]. Briefly, 1 mL reaction mixtures were prepared by adding aliquots of the enzyme (0.14–1.50 μM) to the assay buffer (50 mM TES, pH 7.4, 25 mM MgCl_2_, 6.5 mM HCHO, 1 mM EDTA, 75 mM β-mercaptoethanol) including variable concentrations of dUMP (3–180 μM) and mTHF (5–75 μM). Reactions, started by the addition of the substrate, were monitored by following the increase in absorbance at 340 nm during the oxidation reaction of mTHF to 7,8-dihydrofolate (DHF), for 3 min. K_M_ (Michaelis-Menten constant) values were determined for both mTHF and dUMP by varying their concentration in the assays, whereas k_cat_ was determined by changing the enzyme concentration.

### 2.4. Circular Dichroism (CD) Thermal Denaturation Analysis

The thermal stability of wild-type HT-hTS and the Q62R variant was evaluated using thermal unfolding experiments by monitoring the far-UV CD signal at 220 nm, on a Jasco (Pfungstadt, Germany) J-815 spectropolarimeter. The protein samples (20 µM enzyme solution in 10 mM HEPES pH 7.5 and 100 mM NaCl) were heated from 25 to 75 °C at a rate of 1 °C min^−1^. Two sets of experiments were performed on the HT-hTS and the Q62R variant. The first was performed on the purified proteins, whereas the second on the samples exposed to 1 mM dUMP for 30 min on ice. Measurements were performed in triplicates. Data were analyzed using the software GraphPad Prism 7 in non-linear regression using the Boltzmann sigmoidal function for melting temperature (T_m_) determination.

### 2.5. Protein Crystallization

Prior to crystallization experiments the purified protein was concentrated to 20 mg mL^−1^ and stored at −20 °C (until required). Crystallization trials were performed on the purified HT-hTS Q62R (20 mg mL^−1^ in 50 mM HEPES pH 7.5, 30 mM NaCl, with or without 20 mM β-mercaptoethanol) using the commercially available kits PEG/Ion, Index and Grid screen Ammonium Sulfate from Hampton Research (Aliso Viejo, CA, USA), and JBScreen Basic (JBSB) 1–4 and Classic (JBSC) 6 from Jena Bioscience (Jena, Germany). Protein crystal growth was observed in 10–14 days using the JBSC6 solution C4 (2.0 M ammonium sulfate, 100 mM TRIS pH 8.5) as precipitant.

The optimization of the crystallization condition was performed using the hanging drop vapor-diffusion method [16] at 20 °C, by varying both the ammonium sulfate concentration and the buffer. Crystals, suitable for diffraction experiments, were obtained from drops prepared by mixing equal volumes of protein (above protein solution, with or without 10 mM dUMP) and precipitant (2.0–2.2 M ammonium sulfate, 100 mM bicine pH 9.0) solutions, equilibrated over 800 μL reservoir. Crystal growth was observed within two weeks only in drops prepared by including 20 mM β-mercaptoethanol in the sample solution. Prior to X-ray diffraction experiments, crystals were transferred to the cryoprotectant solution (20% vol/vol glycerol, 2.4 M ammonium sulfate, 100 mM bicine pH 9.0) and flash frozen in liquid nitrogen.

### 2.6. Data Collection, Structure Solution and Refinement

X-ray crystallographic data were collected using synchrotron radiation at the European Synchrotron Radiation Facility (ESRF, Grenoble, France) beamline ID30B, equipped with a Dectris (Baden-Daettwil, Switzerland) Pilatus3 6M detector. Reflections were indexed and integrated using the program XDS [17] and scaled with SCALA [18] from the CCP4 suite [19]. Data collection and reduction statistics are displayed in Table S1. Crystals of HT-hTS Q62R belonged to the primitive orthorhombic space group P2_1_22_1_, including eight enzyme subunits (four enzyme dimers) in the cell asymmetric unit (ASU). The structure was solved by molecular replacement using the software Molrep [20] from the CCP4 suite. One monomer of hTS in the active (PDB id 1HVY [7]) and inactive (PDB id 3N5G [14]) conformations were attempted as searching models (excluding water molecules and non-protein atoms), providing clear evidence that the enzyme crystallized in the active conformation (active conformation: score of 0.775 and wRfac of 0.423; inactive conformation: score of 0.599 and wRfac of 0.548). The structure was refined with Refmac5 [21] from the CCP4 suite using the TLS parametrization [22] in the last cycles of refinement. The optimal partitioning of the polypeptide chains was calculated though the *TLS Motion Determination* web server [23], resulting in twenty continuous segments. The molecular graphic software Coot [24,25] was used for manual rebuilding and modelling of missing atoms. Water molecules were added through the ARP/wARP suite [26] and checked with Coot. Upon completion of the protein model, inspection of the Fourier difference map clearly evidenced the presence of a ligand bound in the active site of all enzyme subunits. The shape of the map indicated that the ligand was a derivative of tetrahydrofolate (THF) modified at position 5 of the pteridine ring. The two THF derivatives 5-formyl-6-tetrahydrofolate (5-FHTF) and 5-hydroxymethyl-6-tetrahydrofolate (5-HMTHF) were alternatively modelled and refined in this site (in all active sites of the four enzyme dimers). Furthermore, sulfate anions and glycerol molecules from crystallization/cryoprotectant solutions were found within both enzyme dimers (seventeen sulfate anions and two glycerol molecules were collectively included in the model). The occupancies of the exogenous ligands were singularly adjusted to values, resulting in atomic displacement parameters close to those of neighboring protein atoms in fully occupied sites. The stereochemical quality of the final model was checked using Coot and Procheck [27]. Refinement statistics are reported in Table S2. Figures were generated through the molecular-graphic software CCP4mg [28].

### 2.7. Protein Data Bank (PDB) Deposition

Atomic coordinates and structure factors for HT-hTS Q62R were deposited in the Protein Data Bank under the accession code 6R2E.

## 3. Results

### 3.1. Variant Production

The hTS Q62R variant was generated through site-directed mutagenesis, using partially overlapping primers and the gene coding sequence for the wild-type enzyme as template for the PCR reaction. In the resulting amplified DNA, the gene coding sequence for the hTS Q62R variant was inserted in the pQE80L vector (plasmid pQE80L—hTS-Q62R), which also included the coding sequence for a non-cleavable N-terminal His^6^-tag. The variant was expressed as His^6^-tag protein (HT-hTS Q62R) in the bacterial strain *E. coli* BL21(DE3). Attempting to express the variant under the same experimental conditions adopted for the wild-type enzyme resulted in the almost complete localization of the target protein in inclusion bodies. To improve the solubility of HT-hTS Q62R, a wide set of expression conditions was screened relying on different incubation temperatures, culture media, inductor concentrations, and incubation times. As expected, the formation of inclusion bodies was generally decreased by reducing the incubation temperature. Indeed, our best conditions turned out to be caused by culturing bacterial cells at 20 °C in the ZYP-5052 auto-induction medium for 48 h.

The purification procedure took advantage from the introduction of the N-terminal His^6^-tag, indeed almost pure (>95%) protein samples for HT-hTS and the Q62R variant were obtained after the first purification step relying on nickel-affinity chromatography. The purification was completed through size exclusion chromatography, resulting in highly pure protein samples (>98%). The final production yield for HT-hTS Q62R was estimated to ~80 mg L^−1^. In contrast, yields of ~200 mg L^−1^ were reported for the wild-type enzyme under standard expression conditions [12,29], and confirmed by us through our modified expression protocol (yield of ~250 mg L^−1^ using the ZYP-5052 auto-induction medium).

### 3.2. Kinetic Characterization

The enzymatic activity assays performed on the HT-hTS and the Q62R variant showed that the point-mutation introduced at the dimer interface perturbs the kinetic properties of the enzyme (Table 1). For the variant Q62R, the K_M_ value for the substrate dUMP is almost unaltered with respect to HT-hTS, whereas the K_M_ value for the cofactor mTHF is ~2.5 times higher. More pronounced is the effect on the turnover rate (k_cat_) that results decreased by more than 6 times in the Q62R variant. This reflects also on the catalytic efficiency, indeed the k_cat_/K_M_ values determined for the variant are 5–17 times lower than those determined for the wild-type enzyme. These results are explained by the binding of a cofactor-analogue molecule to HT-hTS Q62R and by the destabilization effects induced on the dimer quaternary assembly (*vide infra*, Section 3.3 and Section 3.4).

### 3.3. Circular Dichroism (CD) Thermal Denaturation Analysis

**Figure 2:**
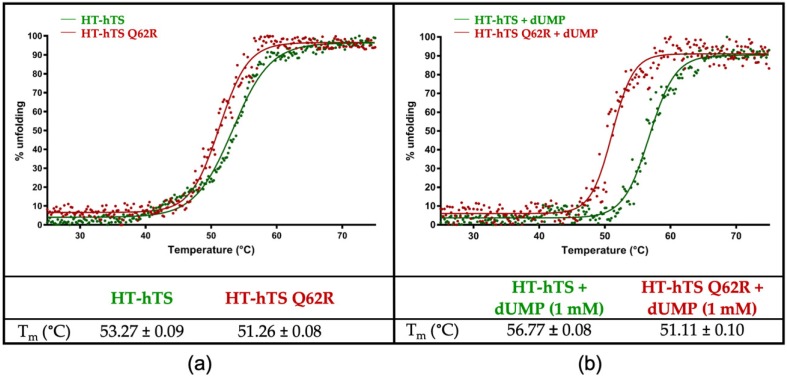
Thermal unfolding transition curves of HT-hTS and HT-hTS Q62R followed by circular dichroism. Two sets of curves were determined, one on the purified proteins (**a**) and the second on the samples incubated for 30 min with 1 mM dUMP (**b**). Melting temperature (T_m_) values determined for the transitions are tabulated in the panels. Measures were performed in triplicate. The thermal denaturation profile of the wild-type HT-hTS suggests a biphasic transition, no longer observed following dUMP addition and in the curves of the variant.

In hTS, residue 62, either a glutamine, in the wild-type enzyme, or an arginine, in the Q62R variant, is exposed at the dimer interface on which it faces the same residue (either Gln62′ or Arg62′) on the cognate subunit. The effect of the introduction of a bulky charged residue at the dimer interface was evaluated through thermal denaturation analysis by monitoring the CD signal at 220 nm (Figure 2). The values of the melting temperature (T_m_) determined for the purified HT-hTS and the Q62R variant resulted of 53.27 (±0.09) and 51.26 (±0.08) °C, respectively (Figure 2a). The decrease by ~2.0 °C in the T_m_ of the variant indicates that the mutation introduced at the enzyme dimer interface induces a destabilization of the protein. The drop in the T_m_ is even more evident after the addition of the substrate (to a 50-fold molar excess with respect to the protein concentration, Figure 2b). At variance with the Q62R variant, for which the same T_m_ (51.11 ± 0.10 °C) was determined after the substrate addition, the wild-type enzyme undergoes to a stabilization effect in presence of dUMP that increases its T_m_ to 56.77 (±0.08) °C (gain by ~3.5 °C). The comparison between the Q62R variant and the wild-type enzyme exposed to the substrate, evidences a reduction by ~5.7 °C in the T_m_ of the interface mutant (Figure 2b). Furthermore, the thermal denaturation profile of HT-hTS suggests a biphasic transition that is no longer observed after the dUMP addition and in the curves of the variant.

### 3.4. Structural Characterization of the HT-hTS Variant Q62R

**Figure 3:**
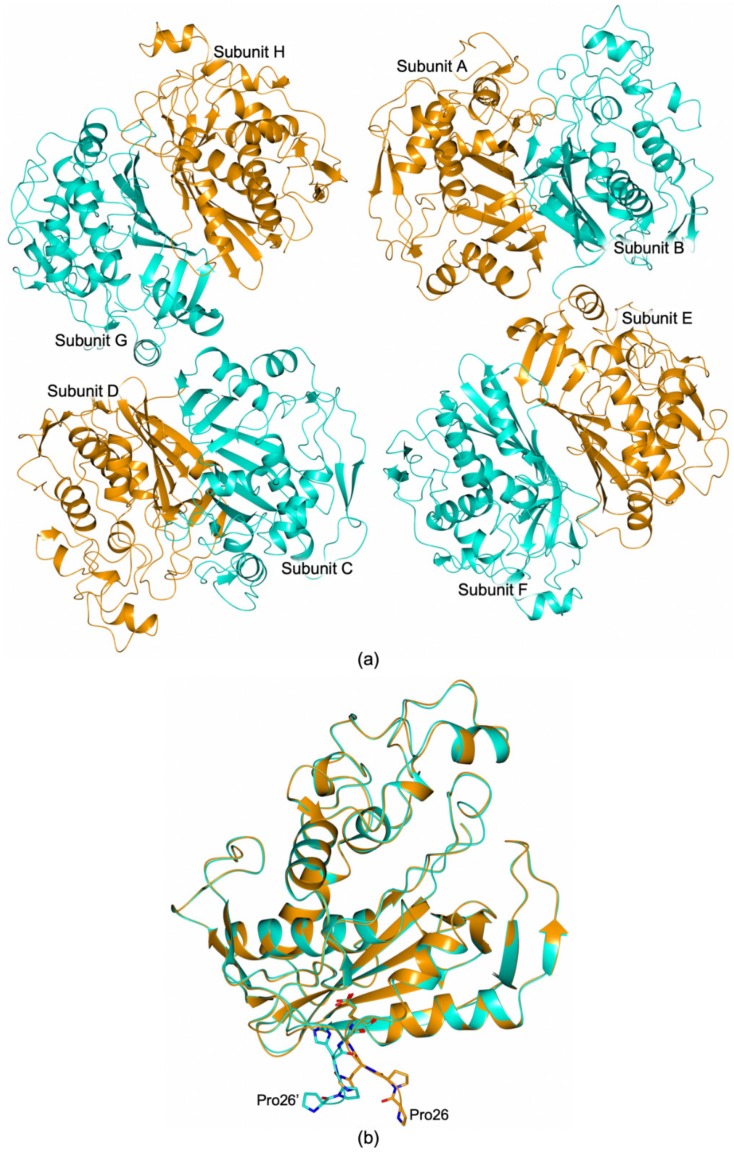
(**a**) Cartoon representation of the four HT-hTS Q62R homodimers (A–B, C–D, E–F, and G–H in our model) found in the cell ASU. (**b**) The structural comparison between subunit A and B (orange and cyan cartoon, respectively) shows that their N-terminal segments point in two distinct directions (residues 26–30 are shown in sticks, carbon atoms are color-coded according to the parent subunit). The maximal displacement, resulting of ~9.5 Å, is observed between Pro26 of two partner subunits (measured between their Cα atoms).

**Figure 4:**
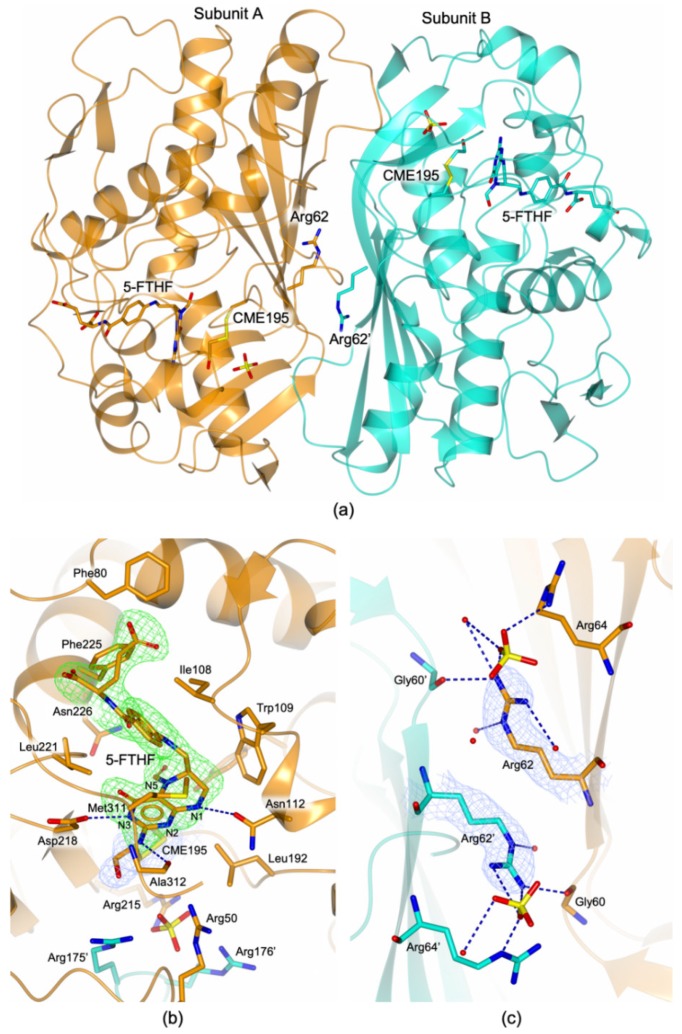
(**a**) Cartoon representation of an enzyme homodimer (subunit A and B are colored orange and cyan, respectively). Within each enzyme homodimer, both subunits assume the active conformation, showing the catalytic Cys195 exposed inside the catalytic cavity. The catalytic Cys195 is modified as *S*,*S*-(2-hydroxyethyl)thiocysteine (CME195, in sticks) in all subunits. The mutated residue Arg62 is displayed in sticks. The cofactor analogue 5-formyl-6-tetrahydrofolate (5-FTHF, in sticks) is entrapped in the active site of all enzyme subunits. (**b**) Active site view of HT-hTS Q62R (in cartoon, interacting residues in sticks; subunit A and B are colored orange and cyan, respectively). The catalytic Cys195 is modified as *S*,*S*-(2-hydroxyethyl)thiocysteine (CME195, in sticks), as visible in the *2F_o_*-*F_c_* electron density map contoured at the 1.5 σ level. The cofactor analogue 5-FTHF (in sticks, orange carbons) is entrapped inside the catalytic cavity by a tight network of H-bonds (blue dashed lines) and van der Waals interactions (water molecules and water mediated interactions have been omitted for clarity). The ligand is surrounded by the omit map contoured at the 3 σ level. 5-FTHF is observed in all active sites of the four dimers found in the ASU. (**c**) Interface view of HT-hTS Q62R (in cartoon, interacting residues in sticks; subunit A and B are colored orange and cyan, respectively). The mutated Arg62 is surrounded by the *2F_o_*-F*_c_* electron density map contoured at the 1.5 σ level. Electrostatic interactions are displayed as blue dashed lines. Water molecules are shown as red spheres. Sulfate anions are displayed in sticks in all panels.

The structure of HT-hTS Q62R was solved to 2.55 Å resolution (Table S1), showing that the mutated protein retains the constitutive dimeric quaternary structure of the wild-type enzyme (Figure 3 and Figure 4a). Four enzyme homodimers (A–B, C–D, E–F, G–H, in our model) were found in the cell asymmetric unit (ASU, Figure 3), all fully traced apart for the first twenty-five N-terminal residues (further the twelve residues belonging to the non-removable His^6^-tag). From the initial phases of structure solution and refinement, it was evident that HT-hTS Q62R was in the active conformation. The four dimers are nearly identical, as testified by the root mean square deviation (rmsd) upon Cα matching that ranged from 0.15 Å to 0.77 Å among all enzyme subunits. The most evident differences are localized in subunits B and D, in which the N-terminal segments point in a distinct direction with respect to other subunits, as evidenced by the structural comparison displayed in Figure 3b. The maximal displacement, resulting in ~9.5 Å, is observed on the N-terminal Pro26 (measured between the Cα atoms).

#### 3.4.1. The HT-hTS Q62R Active Site

The mutation introduced at the enzyme dimer interface did not affect the architecture of the active site, indeed it results fully consistent with formerly reported models for the active conformation of the enzyme [7,13]. Nonetheless, the analysis of the electron density in the active site area evidenced that the catalytic Cys195 was modified as *S*,*S*-(2-hydroxyethyl)thiocysteine (CME195, Figure 4b) by the reaction with β-mercaptoethanol, added to the protein sample prior to the crystallization experiments. The presence of this reducing agent was found to be critical for protein crystallization, indeed attempting to crystallize HT-hTS Q62R without β-mercaptoethanol (or with a different reducing agent) invariantly failed in crystal growth (as formerly observed also for the wild-type enzyme, unpublished results). In all subunits, a sulfate anion was observed nearby the catalytic cysteine, anchored to the guanidinium moieties of the four arginine residues Arg50, Arg215, Arg175′, and Arg176’ (the last two from the partner subunit, Figure 4b). These arginines are also responsible for the recognition of the dUMP phosphate moiety in the substrate binding site [7,13], mimicked here by the sulfate anion. Furthermore, in all active sites, the presence of a folate-like molecule was systematically observed (Figure 4a,b). The shape of the electron density evidenced a bent conformation of the pyrazine ring of the folate pteridine moiety peculiar to the cofactor reduced form, strongly suggesting that the ligand was a tetrahydrofolate (THF) derivative (Figure 4b). The substituent on the pteridine C5 was a bi-atomic species, consistent with either a hydroxymethyl moiety or a formyl group (5-ethyl derivatives of the cofactor are not known). The ligand was refined (in all active sites of the four dimers) either as 5-hydroxymethyl-6-tetrahydrofolate (5-HMTHF) or 5-formyl-6-tetrahydrofolate(5-FTHF), without meaningful changes in the refinement quality indicators (as expected) and in the resulting Fourier maps. Moreover, the resolution of the structure (2.55 Å) did not allow us to distinguish between single and double C-O bonds, preventing further speculations on which THF-derivative is observed in this site. Nonetheless, we opted for 5-FTHF because this molecule is naturally formed inside cells [30] and its complex with the bacterial *Enterococcus faecalis* TS (*Ef*TS, PDB id 3UWL) was formerly reported [31]. The observation of 5-FTHF is further suggested by the orientation, in all enzyme subunits, of the 5-formyl oxygen that is not engaged in intramolecular interaction with the adjacent carbonyl of the ketone group on the reduced pteridine. At variance with 5-FTHF, the presence of 5-HMTHF inside cells has never been detected [30]. We have attempted to refine the putative 5-HMTHF, just for the sake of completeness.

Within the HT-hTS Q62R active site, the cofactor analogue entails a tight network of H-bonds and van der Waals interactions (Figure 4b, only direct H-bonds are shown, water mediated interactions are omitted for clarity). The ketone moiety on the reduced pteridine ring forms water mediated interactions with Asp218, Asn226, and Gln214. Furthermore, the carboxylate moiety of Asp218 is positioned ~2.9 Å away from the pteridine nitrogen N3, strongly suggesting that it is protonated, while donating a H-bond to the protein residue (Figure 4b). The amine moiety on the reduced pteridine forms either direct or water mediated interactions with Ala312 and Asp218 (only the direct H-bond with Ala312 is shown in Figure 4b). The pteridine nitrogen N1 donates a H-bond to Asn112 (Figure 4b). Nearby, the nitrogen N2 forms water mediated interactions with the same Asn112 and with Arg50 (not shown in Figure 4b). Furthermore, 5-FTHF forms close van der Waals contacts with Ile108, Trp109, Leu192, Leu221, Phe225, and Met311.

#### 3.4.2. The Arg62 Pocket

Residue 62 is localized at the periphery of the dimer interface in which it faces itself on the cognate subunit (Figure 1b). The shape of the electron density surrounding residue 62 clearly indicated the presence of an arginine in this site (Figure 4c). At the dimer interface, the side chains of the two facing Arg62 and Arg62′ are oriented in two opposite directions (Figure 4c). This arrangement is adopted to reduce the electrostatic repulsions induced by the presence of two facing charged residues. In this configuration, both arginines interact with the backbone carbonyl of Gly60 on the partner subunit and with various water molecules (Figure 4c). Furthermore, on the two dimer halves, Arg62 is directed towards Arg64 (belonging to the same subunit), creating a positively charged pocket on the enzyme surface in which a sulfate anion (deriving from the crystallization solution) is bound (Figure 4c). The distances separating the Cα of the two facing Arg62 ranged from 8.05 Å to 8.20 (±0.46) Å in the four enzyme dimers found in the ASU.

## 4. Discussion

### 4.1. 5-FTHF Binding in the HT-hTS Q62R Active Site

Former structural studies performed on hTS invariantly reported the yield of the enzyme in the inactive conformation in crystals grown under high-salt crystallization conditions (using precipitant solution including 1.0–1.4 M ammonium sulfate) [7,14,32]. In contrast, HT-hTS Q62R crystallized in the active conformation using a precipitant solution including a higher concentration of ammonium sulfate (2.0–2.2 M). The active conformation adopted in the crystal by HT-hTS Q62R can be ascribed to the population of the cofactor site by 5-FTHF (the formation of the analogous complex in the wild-type enzyme has never been reported). However, the point-mutation introduced at the dimer interface is unlikely to affect the ligand binding since Arg62 is localized more than 15 Å away from the catalytic cavity. Nonetheless, long-range effects have been observed in other enzymes such as *E. coli* class Ia ribonucleotide reductase [33]. On the other hand, the presence of 5-FTHF in the Q62R variant, contributes to explain the observed increase in the K_M_ of mTHF (Table 1), suggesting that the reduced affinity of the cofactor for its site is due to the binding of the ligand. The increased K_M_ measured for mTHF in the Q62R variant respect to HT-hTS indicates competition between cofactor and 5-FTHF, consistently with the crystallographic observation of the enzyme being in the active conformation with both subunits occupied by the cofactor analogue. The binding of 5-FTHF contributes also to explaining the reduced catalytic efficiency of the variant, together with the dimer destabilization effect induced by the mutation.

**Figure 5:**
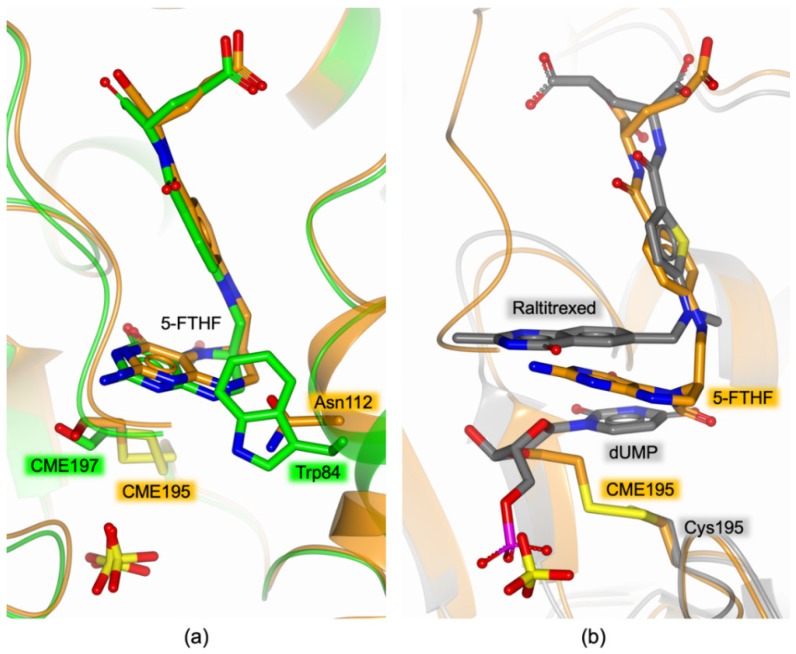
(**a**) Active site view of the superimposition between the structures of HT-hTS Q62R (orange cartoon and carbon atoms) and the bacterial *Enterococcus faecalis* TS (*Ef*TS, green cartoon and carbon atoms; PDB id 3UWL [31]). In both structures, the active site of the enzyme is populated by the cofactor analogue 5-formyl-6-tetrahydrofolate (5-FTHF, in sticks), showing a conserved binding mode. In both complexes, the catalytic cysteine is modified as *S*,*S*-(2-hydroxyethyl)thiocysteine (CME195 and CME197, in HT-hTS Q62R and *Ef*TS, respectively; in sticks). Sulfate anions are shown in sticks. (**b**) Active site view of the superimposition between the structures of HT-hTS Q62R (orange cartoon and carbon atoms, sulfate anion in sticks) in complex with 5-FTHF (in sticks) and the wild-type hTS (grey cartoon and carbons) in complex with 2′-deoxyuridine-5′-monophosphate (dUMP) and raltitrexed (both in sticks). The reduced pteridine moiety of 5-FHTF is moved with respect to the corresponding moiety of raltitrexed, protruding in the substrate uracil site. In the structure of HT-hTS Q62R, the catalytic cysteine is modified as *S*,*S*-(2-hydroxyethyl)thiocysteine (CME195, in sticks).

The wt-hTS—5-FTHF adduct in not available for comparison, but the complex of 5-FTHF with the bacterial *Ef*TS (PDB id 3UWL) has been previously characterized [31]. The comparison with HT-hTS Q62R shows that the ligand adopts the same pose in both enzymes (Figure 5a). Indeed, the active sites of *Ef*TS and hTS are widely conserved, the main difference being on the hTS residue Asn112 that is replaced by Trp84 in the bacterial enzyme (Figure 5a). Even so, both residues are involved in interactions with 5-FTHF, relying on the formation of either a H-bond in HT-hTS Q62R or van der Waals interactions in *Ef*TS (Figure 5a). In both structures, the catalytic cysteine (either Cys195 in HT-hTS Q62R or Cys197 in *Ef*TS) are modified by the reaction with β-mercaptoethanol added during the protein purification/crystallization procedures [31]. It is worth noting that in the structure of HT-hTS Q62R (and *Ef*TS), 5-FTHF populates the cofactor pocket independently by the binding of the substrate. To date all the structures reported for hTS in complex with cofactor analogue inhibitors have been determined in presence of the substrate that populates its site (ternary complexes) [7,13,34,35,36]. Our attempts to characterize HT-hTS Q62R in complex with the substrate dUMP have been unsuccessful. This is reasonably explained by the >200 times higher concentration of sulfate anions present in the precipitant solution that compete with the substrate for the population of the phosphate recognition pocket. Indeed, sulfate anions are observed in this site in all enzyme subunits (Figure 4b). The comparison with the structure of the ternary complex hTS-dUMP-raltitrexed (PDB id 5X5Q [13]) evidences a somewhat different arrangement of 5-FTHF and raltitrexed within the cofactor site (Figure 5b). In the structure of the Q62R variant, the reduced pteridine moiety of 5-FTHF is shifted towards the substrates site, hindering the uracil binding pocket.

### 4.2. Effect of the Interface Point Mutation Q62R on the hTS Dimer Stability

Human thymidylate synthase works as an obligate dimer, showing a stable quaternary assembly due to an extended inter-subunit interface. Indeed, the analysis of the dimer interface through the PISA webserver (http://www.ebi.ac.uk/pdbe/prot_int/pistart.html [37]) results in an average interface area of 2120.8 Å^2^ and a Δ^i^G of −20.4 kcal mol^−1^ (Δ^i^G, indicates the solvation free energy gain upon formation of the interface, calculated on the PDB id 5X5Q [13]). The same analysis performed on the structure of the Q62R variant shows a slight reduction of the interface area, resulting in 2032.0 Å^2^, and a Δ^i^G of −18.7 kcal mol^−1^, suggesting that the introduced mutation induces a destabilization of the enzyme quaternary assembly. The negative effect on the enzyme stability is supported by the results obtained from thermal denaturation analysis. Indeed, for the Q62R variant, a drop in the T_m_ of ~2.0 °C is observed with respect to the wild-type, that increases to ~5.7 °C following the addition of the substrate dUMP (Figure 2). At variance with the Q62R variant, the wild-type enzyme is stabilized by the substrate, showing a gain in the T_m_ by ~3.5 °C (Figure 2). Former studies on hTS demonstrated that, in presence of the substrate, the active/inactive equilibrium (normally occurring in solution) shifts towards the active conformation in which dUMP is observed to bind [7,13]. Thus, the stabilization effect induced by dUMP on the wild-type enzyme is ascribed to the switch of hTS in the active conformation. Furthermore, the thermal denaturation profile observed for the wild-type HT-hTS, suggests that it occurs as a biphasic transition (Figure 2a), due to the active/inactive equilibrium, whereas a monophasic transition, due to the switch of the enzyme in the active conformation, is visible after the addition the dUMP (Figure 2b). Our results confirm those formerly obtained by Chen et al. through differential scanning fluorimetry analysis [13]. On the other hand, an analogous effect is not observed for the Q62R variant for which the same curves have been determined regardless the presence of the substrate. The behavior of the variant is explained by the structural characterization of the “as prepared” HT-hTS Q62R showing that the enzyme adopts the active conformation, stabilized by the interaction with 5-FTHF, that populates the active site. Even though the dUMP binding in the HT-hTS Q62R active site is allowed (indeed the variant is catalytically active, Table 1), the enzyme is already shifted in the active conformation (by the interaction with 5-FTHF), minimizing the stabilization effect induced by the dUMP addition.

**Figure 6:**
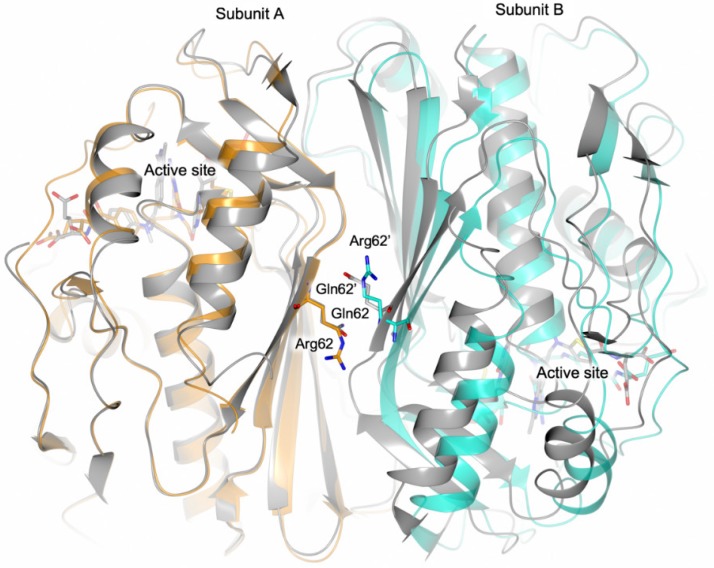
Structural comparison between the homodimer of HT-hTS Q62R (subunit A and B are colored orange and cyan, respectively) and the wild-type enzyme (in grey; PDB id 5X5Q [13]). The superimposition (performed by matching subunit A of both models) indicates a shift of the cognate subunit in the structure of the Q62R variant.

The CD thermal denaturation experiments do not provide direct evidence about the structural reason of HT-hTS Q62R destabilization. However, the comparison of the mutant crystal structure with that of the wild-type enzyme in the active conformation suggests a likely explanation for the observed HT-hTS Q62R destabilization occurring in solution. The superimposition, displayed in Figure 6, provides clear evidence that the point-mutation Q62R induces a slight aperture of the enzyme dimer which is particularly visible in the area surrounding Arg62 and Arg62′ on the two enzyme halves. In the Q62R, a distance of ~8.1 Å is measured between the Cα of the two facing Arg62, whereas Gln62 and Gln62′ in the wild-type enzyme are placed ~6.6 Å apart. The local shift, by ~1.5 Å, is spread over a large interface area (Figure 6), inducing a weakening of the dimer quaternary assembly of the Q62R variant. This is also consistent with the slight reduction of the interface area observed by PISA analysis. The drop (5.7 °C) of the T_m_ of the variant is reasonably explained by the destabilization of the enzyme quaternary structure indicated by the increased inter-subunit distance induced by the mutation.

To confirm this explanation, we will attempt to perform FRET and NMR experiments in solution on the Q62R variant to ascertain the influence of this mutation on the monomer/dimer equilibrium (i.e., determination of the K_d_ and of the equilibrium shift towards the monomeric form).

## 5. Conclusions

Protein-protein interfaces (PPIs) are fundamental for the acquisition of the quaternary structure and for the interaction with partner proteins, but the development of interface targeting inhibitor is difficult. Even though the extension of the interface area is a challenge, the introduction of single-point mutation can induce meaningful perturbations that are exploitable for the development of interface-perturbing drugs. Obligate homodimer enzymes, as hTS, represents a special class of PPIs where the disruption of interface interactions abolishes their catalytic activity. Thus, the hTS dimer interface represents an attractive targetable area for the development of innovative hTS inhibitors. Here, we have investigated the hTS variant Q62R in which the interface residue Gln62 has been replaced by a bulkier charged arginine. The effect of the point mutation has been evaluated through kinetic analysis, CD thermal denaturation studies and X-ray crystallography, providing evidence that the mutation Q62R induces a destabilization effect on the enzyme dimeric structure. Furthermore, our results support the importance of this interface area for the dimer quaternary assembly, in agreement with former studies highlighting the key contribution provided by the nearby residue Phe59 [12]. Indeed, a slight aperture of the hTS dimer is observed subsequently to the integration of Arg62 at the periphery of the inter-subunit interface. The generation of homodimers having a slightly opened dimeric structure, such as the Q62R mutant, can facilitate the access of small molecules to the interface area, simplifying the screening of interface-targeting molecules. Validation procedures on homodimers of the wild-type enzyme are required to verify the effectiveness of the interface-directed molecules and to avoid false-positive binders (e.g., Arg62-interacting molecules). Thus, the hTS Q62R variant, may represent a functional tool exploitable to identify innovative interface-targeting inhibitors.

## Figures and Tables

**Table 1:** Kinetic characterization of His^6^-tag human thymidylate synthase (HT-hTS) and of its interface variant Q62R.

	K_M_ (dUMP) (μM)	K_M_ (mTHF) (μM)	k_cat_ (s^−1^)	k_cat_/K_M_ (dUMP) (μM^−1^ s^−1^)	k_cat_/K_M_ (mTHF) (μM^−1^ s^−1^)
HT-hTS	10 ± 1	6 ± 1	1.00 ± 0.01	1.00 × 10^−7^	1.67 × 10^−7^
HT-hTS Q62R	8 ± 1	16 ± 1	0.16 ± 0.06	0.20 × 10^−7^	0.10 × 10^−7^

## Supplementary Materials

The following are available online at https://www.mdpi.com/2218-273X/9/4/134/s1, Figure S1: Scheme for the TS catalyzed reaction and chemical structures of substrate and cofactor. Figure S2: Active site view of hTS. Table S1: Data collection and processing, Table S2: Structure solution and refinement.
