# Supplementary material for: Evidence of Destabilization of the Human Thymidylate Synthase (hTS) Dimeric Structure Induced by the Interface Mutation Q62R
Source: Biomolecules. 2019 Apr 3;9(4):134. doi: 10.3390/biom9040134 (PMC6523895; doi:10.3390/biom9040134)
Supplement: Supplementary file 1 [file biomolecules-09-00134-s001.pdf]

Evidence of destabilization of the human thymidylate synthase (hTS) dimeric structure induced by the interface mutation Q62R

Cecilia Pozzi[1,*], Ludovica Lopresti[1], Matteo Santucci[2], Maria Paola Costi[2] and Stefano Mangani[1,*]

[1] Department of Biotechnology, Chemistry and Pharmacy – Department of Excellence 2018-2020, University of Siena, via Aldo Moro 2, Siena, 53100, Italy; pozzi4@unisi.it (C.P.); lopresti4@student.unisi.it (L.L.); stefano.mangani@unisi.it (S.M.)

[2] Department of Life Sciences, University of Modena and Reggio Emilia, Via Campi 103, Modena, 41125, Italy; matteo.santucci86@gmail.com (M.S.); mariapaola.costi@unimore.it (M.P.C.)

* Correspondence: pozzi4@unisi.it (C.P.); stefano.mangani@unisi.it (S.M.); Tel.: +39-0577-232132 (C.P.); Tel.: +39-0577-234255 (S.M.)

Table of contents:

Figure S1.	S2
Figure S2.	S3
Table S1.	S4
Table S2.	S5

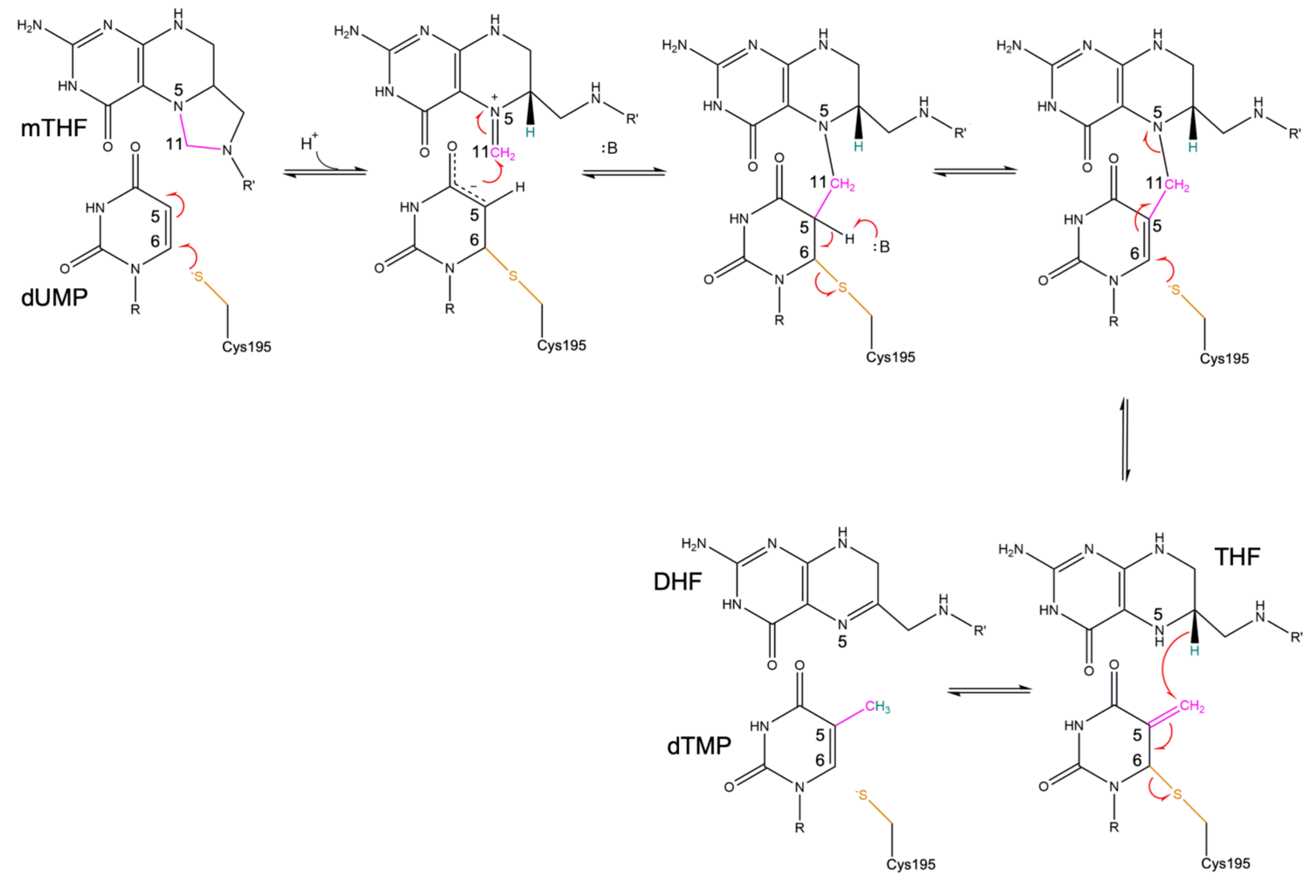

(a)

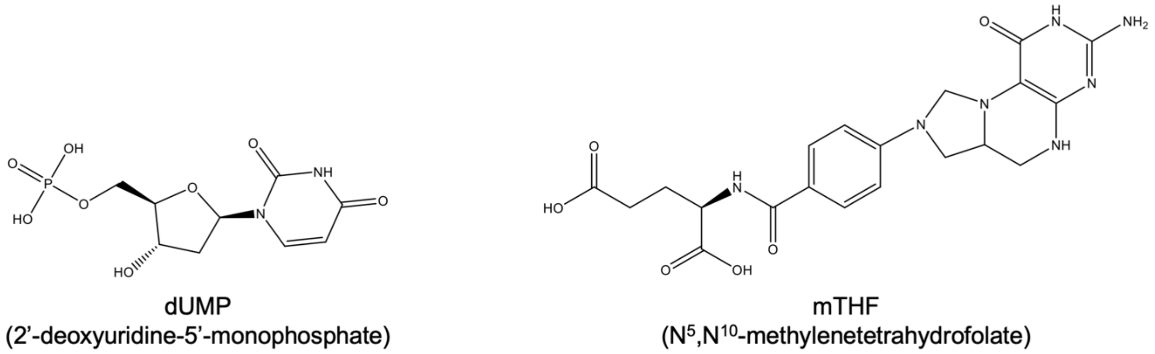

(b)

Figure S1. (a) Proposed reaction scheme for the TS catalyzed reaction [1]. (b) chemical structure of the substrate dUMP and the cofactor mTHF.

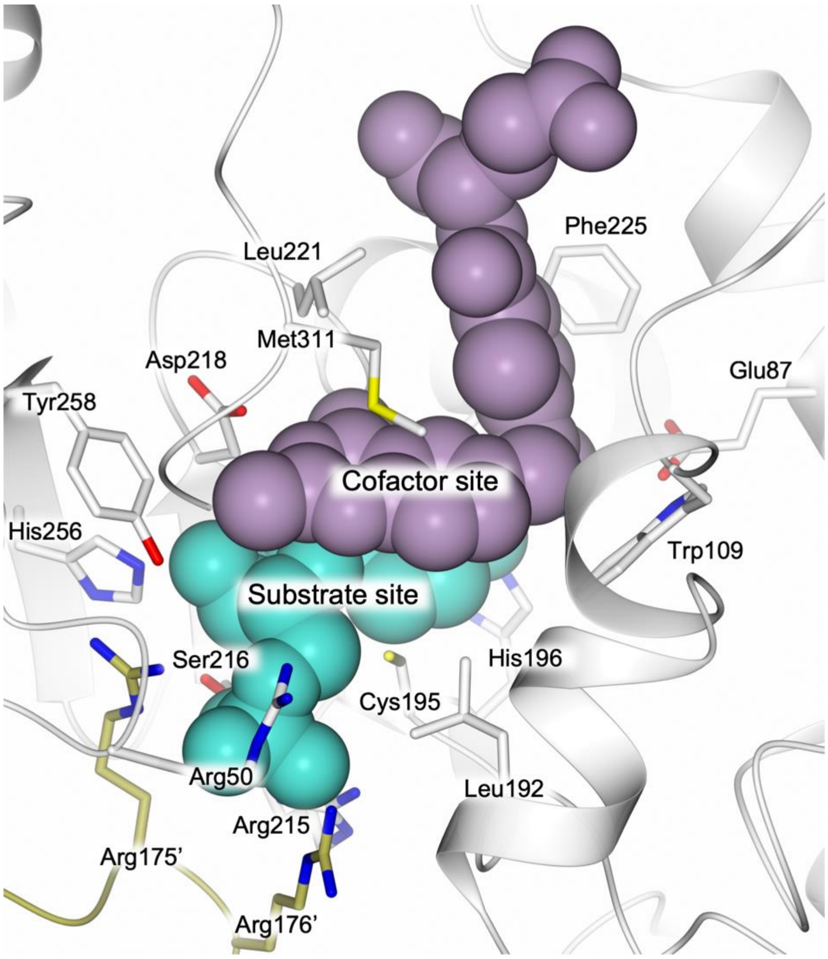

Figure S2. Active site view of hTS (subunit A white cartoon and carbon atoms; subunit B gold cartoon and carbons). Residues are displayed in sticks. The substrate and cofactor site are displayed as turquoise and lilac surfaces. The image has been generated using a structure of hTS in complex with the substrate dUMP (turquoise spheres) and a cofactor-analogue (lilac spheres) (PDB id 1HVY [7]) since a structural model of hTS in complex with the substrate and the cofactor is not available.

Table S1. Data collection and processing (values for the outer shell are given in parentheses).

	**HT-hTS variant Q62R**
**PDB code**	**6R2E**
Diffraction source	ESRF ID30B
Wavelength (Å)	0.96861
Temperature (K)	100
Detector	Dectris Pilatus3 6M
Crystal-detector distance (mm)	460.0
Rotation range per image (°)	0.25
Total rotation range (°)	200
Exposure time per image (s)	0.20
Space group	$P2_12 2_1$
a, b, c (Å)	139.94; 167.07; 189.97
Mosaicity (°)	0.73
Resolution range (Å)	94.99-2.55 (2.69-2.55)
Total No. of reflections	861677 (109995)
No. of unique reflections	145044 (20911)
Completeness (%)	99.9 (99.6)
Redundancy	5.9 (5.3)
⟨I/σ(I)⟩	6.9 (2.1)
R_{meas}	0.178 (0.782)
Overall B factor from Wilson plot (Å^2)	26.83

Table S2. Structure solution and refinement (values for the outer shell are given in parentheses).

	**HT-hTS variant Q62R**
**PDB code**	**6R2E**
Resolution range (Å)	95.17-2.55 (2.62-2.55)
Completeness (%)	99.79 (99.51)
No. of reflections, working set	137694 (10032)
No. of reflections, test set	7182 (554)
Final R_{cryst}	0.196 (0.315)
Final R_{free}	0.258 (0.389)
Estimated error on coordinated based on R value (Å)	0.326
No. of non-H atoms	
Protein	18550
Ion	93
Ligand	284
Water	2430
Total	21357
R.m.s. deviations	
Bonds (Å)	0.012
Angles (°)	2.167
Average B factors (Å^2)	44.01
Ramachandran plot	
Most favoured (%)	96.5
Allowed (%)	3.5
